# The Scriptural Economy, the Forbes Figuration and the Racial Order: Everyday Life in South Africa 1850–1930

**DOI:** 10.1177/0038038515570146

**Published:** 2015-10

**Authors:** Liz Stanley

**Affiliations:** University of Edinburgh, UK

**Keywords:** everyday life, letters, racial order, scriptural economy, social change, South Africa

## Abstract

Social change and large-scale transformations are as important to everyday life sociology as to macro sociology approaches. South Africa has been a ‘hotspot’ of change with a number of such transitions occurring in a condensed time-period, in particular regarding ‘race’ matters. A large South African family collection, concerning the Forbes family, is used to explore how the processes of change regarding the racial order can be analysed within an everyday sociology framework, focusing on the period 1850 to 1930. A range of documents throwing light on ‘the space of the day’, ‘the world and the word’ and other aspects of everyday experience are discussed.

## Social Change, Everyday Life and the Forbes Figuration

It is a truism that Sociology is concerned with explaining the processes of change, a concern embedded in such concepts as modernity, late modernity, postmodernity and globalisation (Dürrschmidt and Taylor, 2007; Heaphy, 2007; Perrons, 2004). Indeed, it has been proposed that sociological attention to change is so central that historical sociology should be seen, not as an area of specialism but, in Philip Abrams’ (1982: 191) phrase, ‘the essence of the discipline’ (Lachmann, 2013; Sewell, 2005).

From this viewpoint, for sociological explanations to ‘work’ they need to get to grips with how change happens. And while the main focus of disciplinary attention has been at the macro-level and large-scale transformations such as industrialisation, capitalism, urbanisation and migration, the smaller-scale focus of the interactional and everyday life sociologies is no less concerned with such matters, not least because telling about the social world and its past, present and future necessarily engages with time and change (Atkinson and Housley, 2003; Highmore, 2001, 2011; Scott, 2009). An example connected with one such transformation – migration – helps convey the ‘world in a grain of sand’ character of the small-scale.

David Forbes and brothers Alexander and James, members of a white Scottish family, migrated in 1850 to Natal, South Africa; his sisters Lizzie and Jemima, in secure work as a housekeeper and ‘superior’ servant, remained in Scotland. Working initially as a labourer and trader, on marriage he turned to farming for a commercial market at Doorn Kloof, Natal. Also, through an emigration company he purchased farms in the Ermelo district from the Transvaal government; he and his family moved to one, Athole, in 1869, leasing the others. His brothers lived partly at Athole, and partly pursued independent economic interests. He was among the first ‘diggers’ following the discovery of diamonds at New Rush (later Kimberley), building up useful capital. Close connections with his sisters in Scotland continued around many practical exchanges occurring in both directions; also numerous material associations with other households connected by kinship and friendship occurred, including that at Westoe, farmed by his in-laws the Purcocks, producing many exchanges of goods, services, monetary help and the borrowing and lending of the labour of black workers. Forbes and his brother James prospected and bought minerals concessions in Swaziland, some leading to mining companies floated on the stock-market. His sons David (aka Dave) and James (aka Jim), extended the economic fabric in mining and droving. Following Forbes’ death in 1905, the Estate was reconfigured and his daughters Nellie, Kittie and Maggie and wife Kate became important farmers. At points, the web of people linked to these manifold activities numbered many hundreds even excluding shareholders, with over 500 at Athole alone (Bonner, 1982; Crush, 1987; Krikler, 1993).

The papers of the Forbes run from 1850 to 1930, with a tail to 1938.^1^ The collection includes over 20,000 letters between family, friends and business and official connections; there are also many diaries, ledgers, lists and tallies, notes, maps, land records, wills, powers of attorney and more.^2^ The Forbes papers are ‘documents of life’ (Plummer, 2001; Stanley, 2013a) *par excellence*, provide a continuous week-on-week (at points day-on-day) longitudinal record of the everyday lives of the people concerned and were mainly written in circumstances of interrupted presence, rather than long-term more permanent absence.

Norbert Elias’ (2000: 481–482, 2009: 1–3) concept of ‘figuration’ is particularly apposite for conceiving the contents of this collection and South African family collections more generally, for the writers are connected throughout; although none involved at the start are still there when the collections end, the flow of letters and connections is uninterrupted. Thought of in figurational terms, the Forbes collection contents enable a truly Qualitative Longitudinal Research (QLR) (McLeod and Thomson, 2009: 59–69; Stanley, 2013b, 2014) approach, exploring how the people concerned from 1850 to 1930 represented the everyday and its events and rhythms as well as social transitions, and also changes in these representations. South Africa is a microcosm for exploring social transformation and its ‘big questions’, while the Forbes and related collections enable doing so at small-scale, over a lengthy time-period, using very large amounts of qualitative data.

South Africa is an instance of what Jorg Dürrschmidt and Graham Taylor (2007) term ‘hotspots’ of rapid economic, political, urban and cultural change, for it has experienced repeated transitions in a relatively short timespan (Feinstein, 2005; Lester et al., 2000: 311–322; Maylam, 2001). Dürrschmidt and Taylor (2007: vii, 6–7) describe their approach to investigating this as ‘deep drillings’ to produce:

an analysis based on a patchwork of ‘thick descriptions’ of transition … to highlight the complexity of social change and … focus on the ways in which contemporary patterns of social changes are underpinned by the operation of complex, non-linear, dynamic processes.

They provide an attractive strategy for exploring change in South Africa, excepting their focus on ‘contemporary patterns’. Regarding its most recent hotspot transition, 1994 democratic elections and subsequent developments, for instance, this does not make full sense until located in relation to previous transitions, each having their own patchy, non-linear, cumulative dynamics.

This is to think with Charles Tilly (1984, 2006) and *contra* the presentism he sees preventing good sociological analysis of change, and in particular to reject seeing social change as coherent, general and explicable *en bloc*. Tilly instead promotes analyses that are ‘concrete in having real times, places and people as their referents’ (Tilly, 1984: 14). He also comfortingly points out, ‘[i]f the work is historical, it need not be grand’ (1984: 14), and the macro questions concerning social change can be analytically explored through the small-scale and specific. At this point, enter everyday life sociology with its emphasis on the everyday and familiar and using this to address the big questions of sociological analysis (Highmore, 2001, 2011; Moran, 2005; Scott, 2009; Silva and Bennett, 2004).^3^

The everyday and its relationship to ‘bigness’ has been interestingly raised by Shula Marks and Anthony Atmore (1980: 2) regarding South Africa, in emphasising the importance of ‘seeing the nineteenth century as it happened not as it turned out, and… by relating the history of the white man [*sic*] in South Africa to that of the majority of its inhabitants and to the imperial context’; and the same question asked here about the 19th century must be asked of earlier and later ones too. This emphasis on seeing the past ‘as it happened’, not as it turned out, underpins everyday life sociology’s attention to the unfolding ‘as it happened’ character of life in the past.

Generally, temporal aspects are present in everyday life sociology but remain implicit: time passes, but with an emphasis on the incremental ‘now’ of the quotidian, not on how and in what ways the everyday is the locus of transitions of different kinds. There are, however, influential everyday life approaches directly concerned with time and change. For example, Henri Lefebvre’s (2013) analysis of rhythmanalysis concerns the changing rhythms and patterns of the everyday. Also Michel de Certeau’s (1984: xx) interrogation of the practice of everyday life situates this in terms of a ‘control over time’. In addition, Elizabeth Shove et al.’s (2012) analysis of the transformational dynamics of social practices shows that, even in work focused on ‘now’, questions of temporality and change can nonetheless be treated as core.

Everyday life sociology has adopted a variety of investigative strategies. When ‘the past’, especially the far past, is the topic of inquiry, then ‘documents of life’ (Plummer, 2001; Stanley, 2013a) rather than researcher-generated data come into frame. Documents of life can include the visual, oral and material as well as written; they are ‘found’ and ‘naturally occurring’, rather than produced for research reasons; they represent the concerns of their originators and context, rather than fitting a predetermined researcher-designed frame. And importantly, they *are* the past (or rather slices of it) as it happened, not as it turned out. The Forbes collection comes under this heading.

Michael Sheringham’s (2006) strategy for everyday life research focuses on how the everyday is configured, as a temporal and spatial entity. Around this, he pinpoints the figural (sites of practice where key aspects of the everyday become more visible, including ‘the day’ and ‘the street’) and the project (specific practices through which lived experience can be added to figural thinking) as theoretically driven ways of operationalising an everyday life investigation.

The Forbes collection is composed of letters, diaries, ledgers, lists, tallies …. These are the components of what De Certeau (1984: 131–164) calls a ‘scriptural economy’ and its ‘quotations of voices’; and, as a representational order, they differ in kind from the lived experience that everyday sociology is generally concerned with. They are all part of (rather than a commentary on) the everyday; however, they situate audience, the moment of writing, and what can be written and how, differently from each other. They are about ‘the same thing’, the everyday, but represent this in different terms, with each scriptural form having its own conventions.

Thomas and Znaniecki’s (1958 [1918–1920]) *The Polish Peasant in Europe and America*, one of the great proto-everyday life sociological accounts of social change, is based on letter-collections. Their interest is in ‘the letter’ as a dialogical form of exchange which is ‘plastic’ – responsive to changing modes and conventions and also to changing technologies for writing, sending and reading – and with epistolarity thereby acting as an index or proxy for changes occurring ‘outside’ as well as ‘inside’ correspondences. The use of letters and other documents of life in investigating change enables not only QLR research, but also an everyday ‘as it happens’ one, for letters, diaries, lists and so on are written in the moment and without foreknowledge and have an emergent and *in media res* character (Stanley, 2004, 2010, 2011).

## The Scriptural Economy

For De Certeau (1984), writing and its scriptural apparatus is in opposition to but inseparable from the oral. Writing, particularly via its reproduction through printing, situates the oral as repressed ‘voices’ haunting written texts. Central here is writing as a constitutive medium with key elements: a blank page specifying what form of writing is required, a text constituted via the conventions attached to this and an enactment in which the text refers back to a lived reality and claims referential force.

De Certeau (1984: 135) terms this a ‘writing laboratory’ that collects, classifies and transforms. This includes the relationship with speaking, by transforming speakers into textual subjects and remaking the varieties of talk into texts structured by genre conventions. De Certeau also perceives fissures, metaphorical (and sometimes literal) smudges and scribbles that insinuate the oral into the written. He discusses two such, the ‘science of fables’ (written stories originating in oral culture), and the ‘return of voice’ (in plays and opera), seeing stories of the everyday as transformed into text in conformity with its conventions, and speech returning only as quotations of voice.

These points are picked up later. However, what is most notable about the Forbes scriptural economy is not that it contains what is written, but its sheer excess. The Forbes parents and offspring, sisters, brothers, uncles, aunts, cousins, friends, neighbours, business connections, shop owners, merchants, tax inspectors, vets, magistrates and many more wrote, and wrote again.^4^ The letters circulating within South Africa provide greetings and information, but the core purpose is of a performative (Austin, 1962) ‘I send you pig lard, please send me a cartload of mealies [corn]’ kind; they are very much ongoing communications in circumstances of interrupted presence where people will meet again fairly soon and/or where the activities expedited by their letters are ongoing. Thus, although not akin to talk, they *are* the continuation of an active relationship by means proxy to talk. Letters circulating between South Africa and Scotland show surprisingly few differences. There is more relaying of ‘news’ about things known in common to keep the other person up to date, but these exchanges too are marked by their performative character and concern shared activities of a ‘will you go to a share-holders meeting for us’ and ‘I sent you a newspaper with information of interest’ kind. There is also little sense of permanent absence or the mediation of identities of gain and loss that the migrant letters literature considers definitional (Elliott et al., 2006; Gerber, 2006), much more the characteristic shared with the intra-South African letters, of getting on with the shared business in hand.

These largely unschooled practical farming and business folk produced everyday writings on a massive scale (Barton and Hamilton, 1998; Lyons, 2012), with the scriptural economy interconnecting with wider Forbes economic activities. In these everyday writings, the business involved is usually literally business, concerned with expediting their shared economic interests; and they are characterised by exteriority (things, people, activities) rather than interiority, and performativity rather than reflection or retrospection. And while it is not surprising that ledgers, lists and tallies would have such characteristics, they also mark the many Forbes diaries, which rarely mention anything ‘personal’ or self-fashioning. These typically describe the working day, its tasks and divisions of labour between different groups of workers and family members; weather patterns; and measures of high/low temperature, wind and rainfall; only occasionally are ‘outside world’ matters mentioned. They are in fact farming diaries pertaining to linked economic entities: the homefarm, Athole Estate, linked farms and their households.

De Certeau’s (1984) ideas about the scriptural economy and its relationship to ‘voice’ raise interesting issues concerning how these Forbes everyday writings are to be understood vis-a-vis each other. Jack Goody (1977: 77) points out that writing not only permits communication across time and space but also re-orders and thereby re-defines meaning through its acts of translation. The list is an important exemplar, for it ‘relies on discontinuity rather than continuity … can be read in different directions … encourages the ordering of the items … [and] brings greater visibility to categories, at the same time as making them more abstract’ (Goody, 1977: 81). Some aspects of this are shared with other kinds of writing. But while re-ordering is strongly characteristic of lists, and less so of tallies (counts) and incomings and outgoings in ledgers, these latter give as much emphasis to the categorical as a means of organising information. In addition, while letters and diaries are outside the parameters of Goody’s discussion, the form they take in the Forbes scriptural economy has similar characteristics. Many Forbes letters have sandwiched between address and signature what are in fact lists and tallies, tables and inventories, as well as sometimes enfolding banknotes, maps and other items. And given their structure, the diaries can be read from bottom up, from temperatures and so on, through weather variations, to the working day; and their measures have definite list features.

These writings also share a key feature: their ontological basis as a representational order. They do not provide access to everyday lived experience, but are written translations that follow the emergent mediations of genre codes. But they *are* a representational order; and the practices of this writing laboratory *can* be engaged with in methodologically coherent ways. At this point, returning to Sheringham’s (2006) ideas for operationalising everyday life research helps give shape to investigating these practices.

## The Writing Laboratory: The Figure and the Project

Sheringham’s (2006) thinking about the figure and the project provides a theoretically grounded basis for research design. So what figural aspects of the Forbes scriptural economy are comparable to the day and the street? In deciding this, the emphasis in what follows is on the unfolding racial order and how people and activities are represented in the practices of this particular writing laboratory.

### The Space of the Day

The first figural device follows ‘the space of the day’. The day is 3 May 1909, selected by searching across the collection for writings with a common date (transcriptions of documents discussed will be found at http://www.whiteswritingwhiteness.ed.ac.uk/action-research/). This produced 16 instances of multiple items with a common date, with this particular day chosen randomly. Given the size of the collection, the low number may seem surprising. However, although most diary-entries are dated, as are many letters, there are still many letters without years or no dating at all, and a small minority of ledger-entries and even fewer notes and lists are dated.

The post was taken and collected daily by one of the Forbes’ workers. The 3 May 1909 letter is a formal one from the Resident Magistrate at Ermelo, the district centre, providing a receipt for a cheque which David Forbes senior had sent for the Manager of the Land [aka Standard] Bank, to pay interest on a loan.^5^ An ordinary letter on one level, it is puzzling why the Magistrate was used as an intermediary, rather than the cheque sent direct. Also, Forbes was a wealthy farmer, owned a number of leased farms and received an income from shares, while the interest paid (£17.10.0) indicates a large loan. And, not ‘there’ on the page but gained from wider knowledge of the collection, the Forbes had two different bank accounts for different purposes.

‘The Forbes’ was both a family unit and a wider economic one with a number of interconnected nodes, with a separate bank account for the family and for the wider Estate. Athole and its related farms were evacuated and seriously damaged during the South African War (1899–1902), and the loan was for rebuilding and re-stocking. Why the Resident Magistrate was an intermediary remains puzzling, however. A routine letter arrived, then, confirming a transaction which points, elliptically, to matters concerning property and a broader entity of the Estate. There is also the question of the space of the day. Written on 3 May 1909, the letter may or may not have been collected and taken to Athole that same day, so 3 May might be either a specific or an approximate temporal guide.

The space of the day takes formal shape in a ledger, which for 3 May 1909 records two cheques drawn on the Standard Bank account.^6^ One is for £5 to ‘SD Straker on account of James Forbes’, and the other for £7.17.0 payable to Macintosh & Kennerley for an antenuptual contract regarding Kitty Forbes’ marriage to Henry Mawson. Sarah Straker was Kate Forbes’ widowed sister, while the Estate of their father David Purcocks was still operative, paying amounts to both. Kate’s son Jim often stayed with his aunt, as various land, cattle, sheep and other livestock was owned and worked in common, with this payment in recompense for board and lodgings and other ‘work’ expenses. Entries on related pages also point to the economic fabric, with amounts for sheep dip and fertiliser. Also the interest paid by David Forbes is recorded against 23 April regarding a £700 loan, with the differently configured day of the letter and the ledger colliding at this point.

The ledger-entries for Straker and Macintosh & Kennerley give a cut and dried view of 3 May 1909: two cheques issued regarding named people and particular goods or services. The surrounding entries have to be searched for signs that most economic activities and transactions involved black people. The preceding entry concerns a cheque for £40 to A. Harper ‘to pay Kafir wages’; it marks the point where the cash economy of amounts paid out and that of paper transactions met, with Harper a white employee who paid wages to Estate workers. The ledger was kept by Kate Forbes. She also managed the homefarm and superintended running the farmhouse. In these capacities, she drew cash sums to pay wages, with many undated lists and tallies recording such amounts. The recipients of these sums are unnamed here, not because of racial homogenising but because recording cash payments lay outside the ledger-entries.

The black majority are represented more in those parts of the scriptural economy closely concerned with the everyday fabric. There are many Forbes diaries, kept variously by David senior, Kate and at points Dave. The diary-entry for 3 May 1909,^7^ by Kate, is atypically structured for a diary-entry and also very different from the letter and ledger. No letters arrive, no cheques are written, no post dispatched. It consists in eight short paragraphs, the last recording temperatures. Five concern events elsewhere: Mr Evans arrived in a motorcar and took Dave to Jim’s farm; Jim and Maggie paid taxes in the local town; Jim and his mother paid money owed to Sarah Straker; a farm worker looked for lost sheep; another went to the police to identify trespassers. Two concern Kate: her refusal to allow a neighbouring farmer to graze his sheep on her foraging-grass, and someone telling her where the trespassers had cut her fences.

The payments to Sarah Straker are not recorded in the ledger, so were paid in cash, and as with the ledger-entry, point to close economic as well as familial links. The Forbes Estate, established before and continuing after David senior’s 1905 death, interfaced after 1899 with the Estate of David Purcocks, in which Sarah, Kate, their brothers and Kate’s sons and daughters all had interests. Jim’s payment refers to hiring labour from Sarah – ‘?Newsteen £8 for the winter’ – and probably concerns the annual cattle and sheep dipping. ?Newsteen is implicitly male and black (indicated by name and that he was hired from Sarah, not in his own right). Jim and Maggie (who managed her brother Dave’s farming interests while he pursued his other economic concerns) had also paid taxes due for two groups of black workers. One concerns ‘taxes for 5 fresh boys’. Almost invariably when ‘boy’ is used in Forbes writings it refers to actual boys, rather than as it became, an insulting reference to adult men. All the Forbes males had long-term friendly relationships with the Swazi royal house, so this may refer to a group sent to work for Jim and him paying the Transvaal labour tax. The other – ‘Jim paid the taxes for his Kafirs’ – refers to hut-tax, a poll tax also levied by the Transvaal. The Forbes annually paid hut-tax for all their workers and so were not affected by a 1903 revolt and withdrawal of labour. The word ‘kaffir’ is now a homogenising and insulting term in South Africa. In the Forbes scriptural economy, it is overwhelmingly capitalised and used of a broad collectivity of people as an ethnic reference.

Two other black people are named in this diary-entry, ?Mobin and Monshie. Implicitly black and male, both were long-term presences at Athole, with jobs done and cash wages to them often appearing in tallies and lists. As with Dave, Jim and Maggie, they are recorded with personal names only, but distinguished by activity and type of familiarity. The other, implicitly white, people who are mentioned are recorded with full names or titles (like Mr Evans).

‘The space of a day’ inscribed across these everyday writings is different. It is a single activity, a letter arriving; two economic transactions, cheques written, and with surrounding references implying black workers; and a diary-entry including black people in not entirely straightforwardly ways. The more formalised and abstract the writing codes and genre conventions, the less black people are represented; and the closer to the lived experience of everyday life the representational order is, the more their presence comes into focus. The projects following pursue such focus.

### Mrs McCorkindale’s Permission, Umquaka’s Voice, Featuring Bismark

Three projects now add signs of everyday lived experience in representational form to the figural stratagem of the day. They are a pass to travel from one place to another, a letter that is more exactly the quotation of voice and a compendium of everyday writings referring to someone. They are discussed regarding particular documents and the practices of the writing laboratory that produced them.

The pass is a note of formulaic kind acting as a quasi-legal document and was written by Kate Forbes and Sarah Straker’s maternal aunt, Mary McCorkindale.^8^ Dated 13 November 1876, it certifies her permission for ‘the bearers 2 Kaffirs’ to travel from Pretoria to Athole to deliver parcels for her. The requirement that all black people were tied to a place of employment and forbidden to move about unless licensed by their employer was a Transvaal legality. Specific personhood of the people concerned was seen as irrelevant to the extent of them being unnamed, reducing them to ‘2 Kaffirs’. Made visible by this particular pass is the material fabric of ‘the post’. On many contemporaneous letters the words ‘per kafir’ appear, with the mechanisms of sending, collecting and delivery carried out by young boys or, as here, when involving longer distances and larger items, older men. Black labour was the dynamism propelling exchange, the liquidity by which communications across time and space were enabled, and the large majority of other productive work carried out.

The pass system of control had longevity. When the Transvaal was annexed by British forces during the South African War in 1900, whites too were required to have passes to move about. This was greatly resented because it was seen as an extension of racial hierarchy to white ethnicities and particularly the Boer population. It was further modified post-war, with particular employers licensed to issue passes to black workers in the area, with Kate and Maggie Forbes issuing, and sometimes refusing to issue, passes through to 1917. In more draconian forms, it persisted up to the 1994 transition.

The pass system was omnipresent in the everyday realities of black people’s lived experiences of the racial order. It operated as a covering law; and as in this 13 November 1876 example, people of specific identities ‘vanished’ and reappeared in licensed simulacra form reduced to a ‘race’/ethnic category.

The second project concerns an eight page letter of 11 July 1886 by Umquaka.^9^ Umquaka was Swazi and from c1871 worked at Athole as nursemaid to the Forbes children. The letter was sent to Kitty Forbes, in Scotland with her parents, and is certainly Umquaka’s letter and authored by her. However, her authorship takes the form of a written ‘translation’ of her speaking, made by a third party, James junior (Jim), with the translation a double one, from voice to writing and from Swazi to English.

Umquaka’s letter points up interesting aspects of her relationship to various of the Forbes. It is familiar and affectionate in expression, referring to ‘your ma’, ‘big Jim’ and so on, and telling off Maggie and Dave for not writing to her or her children. The authority figure across the farms while the Forbes were away was Sarah Straker, who is familiarly as well as formally addressed, with ‘aunt Sarah’ perhaps a point where the writer surfaces in the text and overwrites Umquaka’s authorship.

The letter as a written quotation of Umquaka’s voice is indicated by slips of the pen. It appears in the writer’s variant spellings of Kaffas, Kafass, Kafase, Kabase. It is most clearly indicated when ‘James’, ‘I’ and ‘big Jim’ are confused because Umquaka had spoken the name while the writer struggled to catch up with who she meant and crossed out the first two. She also draws attention to the author/writer relation herself, commenting ‘you know very well I cant write or read’ and noting Sarah Straker and Mr Phillips as possible alternative writers.

Whiteness is present in not entirely favourable ways. The Forbes are clearly Athole’s top dogs and things revolve around them or their proxy, Sarah Straker. At the same time, Umquaka is clear Kitty should not return ‘white’, but nice as she was before, and that not eating properly makes people ‘thin and white’, with nice, fat and black the implied contrasts. How Umquaka’s translated voice positions other black people is also complicated. ‘Kaffirs’ is used generically, regarding two no-show men from Swaziland who were to have helped re-thatch the farmhouse, and people who put out a fire. However, most references to black people in her letter use personal names. These are people in the familiar world of the homestead and the homefarm and include her children Fass, Kafass and Scotia, Truie and his son, and Timfry. More elliptically, there is reference to an unnamed man who damaged Umquaka’s house so she is no longer safe at night and her complaint that no one – referencing Fred, the white farm manager at the time – would repair it for her.

The third project is a compendium of items over a 27 year period which, among other matters, feature Bismark, a long-term worker at Athole: his first appearance in a letter, on 16 July 1889;^10^ two ledger-entries of May 1913 and May 1913;^11^ an undated Athole census;^12^ and his last appearance, in a diary-entry of 14 November 1916.^13^ There is nothing extraordinary here; Bismark stood out because his name took the eye, not any activities he did or events he was part of.

The 16 July 1889 letter is from Sarah Straker to Kate Forbes, then on a visit to Britain around David senior’s business interests there. Sarah was the Westoe farmer rather than her father, with one of her money-making activities pig-farming, a joint Westoe, Athole and ‘reef’^14^ venture. Her nephews Dave and Jim were too squeamish, so ‘I am going to Athole to see about killing the pigs Bismark is at the reef butchering the pigs.’ Bismark was a peasant-farmer at Athole on the homefarm, with the ledger-entry of 16 May 1912 recording that he was due £6.10.0 for an ox Jim Forbes sold for him. Although Bismark was paid wages, with the ledger-entry of May 1913 recording a substantial payment of £5.0.0, he was not straightforwardly a wage-labourer, but paid for work done on the homefarm and wider Athole Estate (see Bundy, 1988 [1979]; Crush, 1987; Krikler, 1993). The (undated but post-1903) census covers three entities – the Estate, the homefarm and the farmhouse kitchen staff – and records some 501 African people. It was produced regarding the payment of hut-tax, levied against the whole unit and not just the male head. Bismark lived and farmed at the homefarm, sub-divided into different areas; he was one of a majority of African men at Athole with just one wife (and two children), in his case perhaps because he was a Christian convert rather than unable to afford lobola (bride-price). The diary-entry of 16 November 1916 mainly details replanting fields belonging to Maggie and Nellie Forbes and records that ‘Bismark finished planting the fields on one side of the road also Nellies field’.^15^ Many white farmers exacted labour without recompense in return for squatting rights; the Forbes and some others paid at the prevailing local rate for wage-labour.

Bismark was an ordinary man going about his life as peasant-farmer on the cusp of wage-labour, receiving wages for the array of farming and related work he did. This included hoeing, planting, mending buildings, painting, operating a mealies planter and repairing different farm machinery, so he was skilled and versatile. In addition he owned oxen (and elsewhere cows and sheep are recorded against his name) and sold them for a market, so he was also successful. Bismark is a familiar figure in the landscape of the Forbes scriptural economy. He remains at a distance, apart from once when Kate Forbes returned from a visit with presents for people, with Bismark receiving a knife, as did Kate’s father. From 1889 to 1916 Bismark is always referred to by name, glimpsed going about his business and that of the Forbes; there is no change of nomenclature and little of activity, except the sub-divisions of farms change and the machinery becomes mechanised, while the labour remains a constant presence.

### The World and the Word

The second figural stratagem is a spatial parallel to temporality and ‘the day’, but conceived in recognition that ‘the street’ does not translate regarding a large but complexly peopled Transvaal farm across the time-period concerned. ‘The world and the word’ is the figural device. ‘The world’ centres on the farmhouse, the homefarm and the Athole Estate, which encompassed the lives of the large majority of the people the census recorded as living there. At the same time, all its denizens, not just the Forbes, were also in figurational (in Elias’ sense) contact with others working on farms elsewhere, in South African and Swazi mines, and in their towns and cities. ‘The word’ concerns the time-travelling words ‘Kafir’ and ‘boy’. At the beginning of the period the scriptural economy covers, these words circulated in South Africa generally to characterise particular collectivities of people by ethnicity and age. Over time they took on negative meaning as homogenised and negatively loaded racial markers.^16^

As noted earlier, ‘Kafir’ appears in many Forbes writings across the 70 year period these cover. It is capitalised and complicatedly indicates ethnicity, being for example always distinguished from Zulus but on occasion encapsulating Swazis. In lower case and used pejoratively, it occurs later, from the late 1870s and almost entirely in letters by James senior and those later still by Dave. Similarly, ‘boy’ is a literal boy and appears in this way in diaries, ledgers, lists and tallies. Later it is capitalised as ‘Boy’, mainly in letters from James senior and occasionally Dave, with its capitalising contradictorily giving it some of the qualities of an ethnic marker while also negativising its meaning. In mining – the source of the negative usage – it arose because particular age-regiments of young boys were sent by their Chiefs to work on the mines for short periods, and was then later used as a diminutive to refer to adult men. The ‘n word’ also appears, in two forms: ‘niger’ as an Africanising of the word (regarding the Niger area) and ‘nigger’ as a more straightforwardly dismissive term. In both forms it is used mainly by James senior, also by Sarah’s husband Joshua Straker from the early 1880s and Dave from the later 1880s.^17^ It is used as a sarcasm by Sarah in a letter of September 1871,^18^ and in an 1894 outburst from David senior about the Swazis as a people,^19^ but never by Kate Forbes or her daughters.

Is the use of racialised terms perhaps less a matter of generation, gender and context, and more one of particular people – James senior and Dave especially – ‘by temperament’? The main economic activity of both men was mining, James as a digger for diamonds, prospector for gold and coal and hands-on mine owner, and Dave as a Swazi mining concessionaire and then manager of a coal mine there. Also Sarah’s reference came at a point when Kate was at New Rush and Sarah had just returned, and David and Straker were both diggers for extended periods there too. Context, then, is more important than may appear at first sight. However, while Dave is the readiest of all the letter-writers to use these words being racialised and becoming racist, at the same time he was also the Forbes closest to black people and constantly kept company with particular black men on the borders of servants and friends. But of course the written has a tricky, less than fully representational relationship with other aspects of the lived life, and the perception of these relationships on both sides remains elusive.

There is also an important difference between the letters and other everyday Forbes writings. ‘Kafir’ and ‘boy’ as used in the ledgers, tallies, lists and so on retain their ‘old’ meaning through to the 1920s. This usage persisted perhaps because of the spare structure of these forms of writing, including the stylised diary-entries, perhaps because the contents refer to the familiar world and known people of Athole and environs. In addition, the plasticity of the epistolary form noted earlier has to be reckoned with, in particular the capacity of letters to register wider changes occurring and act as an index of change.

However, if the name of the scriptural game is change on the one hand, and stasis on the other, and if the representation does not give referential access to what is represented, then how best to interpret and understand what was going on? And what does this imply about everyday life, the racial order and change?

## Coming to Terms: Real Times, Places and People

Whatever the data, ‘now’ is the place of inquiry; and the representational order of everyday writings of the past, the documents of life that are the Forbes lists, tallies, censuses, ledgers, diaries and letters, provide everyday life sociology with difficulties and also opportunities in gaining purchase on the unfolding racial order.

It is difficult not to operate in a mind-set of ‘how it turned out’ and let the conceptual categories of ‘now’ override the emergent, piecemeal, contradictory, character of how it happened, as explored above. It is difficult even when working longitudinally and regarding specific circumstances and people to find clear evidence of what the changes occurring – including the spread of racialised word uses from one context to another – actually entailed in terms of the conduct of people towards each other. And while the landscape of dominating whiteness and its figural ‘Others’ can be seen, it is only with difficulty and in the smudges and scribbles of writing laboratory practices that an implied positive blackness and a more troublesome whiteness come into focus. These marginalia, then, need to become central for an everyday life sociology concerned with the making of the racial order. Umquaka’s complaint, Bismark’s present and David’s outburst raise these complexities and they and other such scribbles can be pursued.

The Forbes documents of life are remaining traces of ‘the past’, which is for all practical purposes now a representational order only. The scriptural economy, however, is not one and indivisible; each document is marked by its moment of writing concerns; and the genres these are part of are shaped by conventions regarding the ‘what and how’ of writing of particular kinds. So is it perhaps a matter of form, with the flexible and indexical character of the epistolary form resulting in letters being closer to ‘the action’ as it unfolds? This is to suggest that because of their seriality and longitudinality they can better get at the processes and mechanisms of change than other everyday writings. But as the glimpses of Bismark over nearly 30 years show, longitudinality and seriality are not reserved for letters and can (and should) be pursued using other sources too – and anyway, as this example indicates, neither axiomatically yield change.

Relatedly, the particular context of the Forbes figuration has importance for coming to terms with the lumpy, piecemeal way that the racial order changed. For this figuration, family, household, economic livelihood, land and other property and alternative means of making income not only intersected but were overlaid; they stretched from the Transvaal, to Natal, Swaziland, London, Scotland; they morphed over time because of life-stage changes; but with the economic fundaments remaining largely intact. There is no easy means of referring to this complex figurational entity, for none of the available conceptual categories stretch far enough (see http://www.whiteswritingwhiteness.ed.ac.uk/forbes-domus-figurations/). Also, particular features of its economic base and mode of production made it if not impervious then resistant to change. This concerns particularly the farms and land in Natal and then Transvaal.

‘New technology’ arrived for the Forbes when they arrived in Natal and it involved the cheap labour of black people and the land they inhabited, land which was abrogated by the settler colonial governments and sold on. Across the tens of thousands of everyday writings in the Forbes collection, most things are done or expedited or communicated by African labour – ‘per Kafir’, as so many documents record. Black labour as the key technology continues in these writings from 1860 and David and Kate Forbes at Doorn Kloof farm, to Kate’s old age at Athole in the late 1910s and death in 1922, to 1930; and it underpinned both a way of life and a mode of production dependent on resident African peasant-farmers.

The figurational entity referred to above certainly changed over time.^20^ However, the factors producing change were marriage, distance moves, life-stage changes, transnational visits, war, death. There is no sign that mechanisation and motorisation or other technological innovations made any great difference to this way of life for the large number of people involved or that its divisions of labour changed significantly. The ox-wagon persisted alongside the steam-engine and motorcar; hand-hoeing and mechanised picking coexisted, done by the same people; and everywhere in the writing laboratory the omnipresence of such daily labour is inscribed. This is why, for instance, Bismark in 1889 through to 1913 is doing very much the same kinds of tasks, for there were the same kinds of tasks to be done and the mode of production of peasant-farmers carrying out labour in return for rights to live on the land had not changed.

The tallies, lists, ledgers and diaries reflect this lack of change to the mode of production on Athole and its environs because these environs are their locus of concern. The letters represent changes to the racial order ‘outside’ because, as well as the farm and environs, they are concerned with people, things and events in a wider world. This included Union of the settler states in 1910 and rapid introduction of racial legislation, including the notorious Land Act of 1913 which disappropriated land rights for African ‘squatters’ on white farms. There are signs that the Forbes resisted its enforcement at Athole, although later its provisions were reinforced and are likely to have played out by the end of the period the collection covers, with ‘scribbles’ on this a project for further investigation.^21^

These wider matters took what seems a patchy and non-linear form at Athole. However, the farm and Estate and the complexly linked extensive entrepreneurial economic interests the Forbes and others in their figuration were part of are not unique among the many white farmers in South Africa over the period from 1850 to 1930. Coming to terms with the complicated relationship of stasis and change, and the disruptions of monolithic teleological narratives, that the Forbes scriptural economy demonstrates is key to understanding the everyday making of the racial order in such settings, and to comparing these with contexts where the racialised changes which occurred are more straightforwardly visible.

## Notes

1. Research on the Forbes Collection is part of the ‘Whites Writing Whiteness’ (WWW) project (ESRC ES J022977/1). See www.whiteswritingwhiteness.ed.ac.uk.

2. Forbes Family Collection, National Archives Depot, South Africa. All archive references are to this.

3. There are close parallels with microhistory; see Magnusson and Szjarto (2013).

4. A central sub-set consists of David Forbes senior, his sisters Lizzie Forbes and Jemima Condie and brothers Alexander and James; his wife Kate, their children Alex junior, David junior, James junior, Nellie, Kittie and Maggie; Kate’s parents David and Anne Purcocks, her sister Sarah and brothers George, Vincent and David; and Kate and Sarah’s maternal aunt Mary McCorkindale and her husband Alexander. The women here wrote as much as the men, *contra* the migrant letters literature suggestion that generally they did not (e.g. Gerber, 2006: 5–10).

5. Box 1/85.

6. Box 39, main ledger, cheques paid out March–May 1909.

7. Box 24, 1909 diary, 3 May 1909.

8. Box 38 miscellaneous.

9. Box 7/229.

10. Box 8/115.

11. Box 40 main ledger.

12. Box 40.

13. Box 28.

14. A mine in Swaziland which Dave managed, also owning farmland there.

15. A Transvaal regulation required a road through all farms.

16. Within the WWW research as a whole, information about how these words are used is being recorded, as well as regarding the Forbes collection.

17. E.g. JS to DF, 9 April 1881, 7/17.

18. SP to KF, September 1871, 7/145.

19. DF senior to DF junior, August 1894, 8/254.

20. For details, see http://www.whiteswritingwhiteness.ed.ac.uk/forbes-domus-figurations/.

21. Things were very different at Dave’s reef farm; see Crush (1987: 179–180).
